# MicroRNA-33b Suppresses Epithelial–Mesenchymal Transition Repressing the MYC–EZH2 Pathway in HER2+ Breast Carcinoma

**DOI:** 10.3389/fonc.2020.01661

**Published:** 2020-09-10

**Authors:** Birlipta Pattanayak, Iris Garrido-Cano, Anna Adam-Artigues, Eduardo Tormo, Begoña Pineda, Paula Cabello, Elisa Alonso, Begoña Bermejo, Cristina Hernando, María Teresa Martínez, Ana Rovira, Joan Albanell, Federico Rojo, Octavio Burgués, Juan Miguel Cejalvo, Ana Lluch, Pilar Eroles

**Affiliations:** ^1^Biomedical Research Institute, INCLIVA, Valencia, Spain; ^2^Centro de Investigación Biomédica en Red de Oncología, Instituto de Salud Carlos III Madrid, Spain; ^3^Department of Physiology, University of Valencia, Valencia, Spain; ^4^Department of Pathology, Hospital Clinico de Valencia, Valencia, Spain; ^5^Department of Oncology, Hospital Clinico de Valencia, Valencia, Spain; ^6^Cancer Research Program, Institut Hospital del Mar d’Investigacions Mèdiques, Barcelona, Spain; ^7^Department of Medical Oncology, Hospital del Mar, Centro de Investigación Biomédica en Red de Cáncer, Barcelona, Spain; ^8^Department of Experimental and Health Sciences, Universitat Pompeu Fabra, Barcelona, Spain; ^9^Department of Pathology, Instituto de Investigación Sanitaria de la Fundación Jiménez Díaz, Madrid, Spain; ^10^COST action CA15204, Brussels, Belgium

**Keywords:** miRNA-33b, EMT, MYC, EZH2, HER2+, breast cancer

## Abstract

Downregulation of miR-33b has been documented in many types of cancers and is being involved in proliferation, migration, and epithelial–mesenchymal transition (EMT). Furthermore, the enhancer of zeste homolog 2-gene (EZH2) is a master regulator of controlling the stem cell differentiation and the cell proliferation processes. We aim to evaluate the implication of miR-33b in the EMT pathway in HER2+ breast cancer (BC) and to analyze the role of EZH2 in this process as well as the interaction between them. miR-33b is downregulated in HER2+ BC cells *vs* healthy controls, where EZH2 has an opposite expression *in vitro* and in patients’ samples. The upregulation of miR-33b suppressed proliferation, induced apoptosis, reduced invasion, migration and regulated EMT by an increase of E-cadherin and a decrease of ß-catenin and vimentin. The silencing of EZH2 mimicked the impact of miR-33b overexpression. Furthermore, the inhibition of miR-33b induces cell proliferation, invasion, migration, EMT, and EZH2 expression in non-tumorigenic cells. Importantly, the Kaplan–Meier analysis showed a significant association between high miR-33b expression and better overall survival. These results suggest miR-33b as a suppressive miRNA that could inhibit tumor metastasis and invasion in HER2+ BC partly by impeding EMT through the repression of the MYC–EZH2 loop.

## Introduction

Breast cancer (BC) is the most frequently diagnosed malignancy among women worldwide and considered as the most threatening cancer for women’s health (1). Breast cancer mortality still accounted for about 25.3 per 100,000 women in 2018 (2). During the recent years, to better understand BC biology, many efforts have been performed, leading to elucidate the heterogeneity of different subtypes [luminal A, luminal B, HER2-positive (HER2+), and triple-negative] susceptible for personalized approach and treatment. HER2 gene amplification occurs in 20–25% of BCs and is associated with disease relapse and poor prognosis. There are different targeted agents; both monoclonal antibody and tyrosine kinase inhibitors have radically changed the history of this disease (3). Nevertheless, after a neoadjuvant or an adjuvant approach, the rate of relapse remains substantially high (4). It is therefore essential to explore deeply the molecular mechanisms responsible for disease progression and therapy resistance to identify possible biomarkers that would guide novel treatments for this subtype of BC.

MicroRNAs are small molecules based on 20–22 nucleotides, having a main function in regulating gene expression post-transcriptionally by inhibiting protein translation or causing the degradation of the target mRNA (5). Different cancer types have a varied expression of miRNAs; in addition, some miRNAs may function as oncogenes or tumor suppressors. They have been directly implicated in cancer metastasis or the prevention of cancer progression by participating in the regulation of the epithelial–mesenchymal transition (EMT) pathway, stemness and targeting apoptosis pathway. In this scenario, miR-33b was found to act as an anti-cancer miRNA, inhibiting cell migration, proliferation, and invasion in melanoma cancer (6), lung cancer (7, 8), prostate cancer (9), osteocarcinoma (10, 11), gastric cancer (12), and triple-negative BC (TNBC) (13). However, the role and the action mechanism of miR-33b in HER2+ BC subtype are still unclear.

Furthermore, the enhancer of zeste homolog 2-gene (EZH2) has a master regulatory function in controlling processes such as stem cell differentiation, cell proliferation, early embryogenesis, and X chromosome inactivation (14). EZH2 is overexpressed in metastatic prostate cancer and promotes cell metastasis and proliferation by inhibiting apoptosis (15). It is also described as a master regulator of the EMT by overexpressing Snail, Slug, and vimentin and suppresses E-cadherin (CDH1) expression in endometrial cancer and gastric cancer (16, 17), but less has been explored in the HER2+ BC subtype. Moreover, the transcription factor MYC (18) has been suggested as a positive regulator of EZH2 by different mechanisms in several types of cancers. MYC might enhance EZH2 expression through inhibiting the microRNAs miR-26a and miR-26b (19) and also by the activation of the EZH2 expression through binding with E-box, a DNA binding site of MYC (20). Emerging shreds of evidence also showed that miR-33b negatively regulates MYC in osteosarcoma cancer (10) and prostate cancer progression (9) by directly binding with its 3′ UTR region. Moreover, there is recent substantial data which suggested that miR-33a could negatively regulate EZH2 in cancer progression by direct interaction in TNBC (21).

Taking all these information together, our paper aimed to explore more about miR-33b from miR-33 family in HER2+ BC. As HER2+ is an aggressive disease with significant mortality, it requires massive molecular mechanism studies to defeat its aggressiveness (22). Our results show, for the first time, that the under-expression of miR-33b is related to the poor prognosis and low survival in HER2+ BC, while a high expression of EZH2 is directly proportional to tumor aggressiveness and proliferation. As the miR-33b and EZH2 molecular mechanism functions have been less elucidated in this subtype of BC, we tried to fill the loophole between them. MiR-33b exerts its function by indirectly targeting EZH2 through directly inhibiting MYC to repress the migration, invasion, proliferation, and EMT development of HER2+ BC. Furthermore, we identified a novel 33b/MYC/EZH2 axis implicated in proliferation and invasion in HER2+ BC.

## Materials and Methods

### Cell Culture and Reagents

Human BC cell lines BT474, SKBR3, MDA-MB-468, MCF7 and MCF-12A, and MCF-10A non-tumorigenic epithelial cells were maintained in Dulbecco’s modified Eagles medium (GIBCO) supplemented with 10% fetal bovine serum (FBS; Gibco), 10,000 U/ml penicillin, 10,000 μg/ml streptomycin, and 1% L-glutamine (200 mM) (×100). All cells were cultured at 37°C in 5%-CO_2_ atmosphere.

### Transfection

The cell lines were transfected either with 100 nM hsa-miR-33b-5p mirVana mimic (assay ID MC12289, Ambion) or inhibitor miRNAs (assay ID MH12289, Ambion) and 100 nM EZH2 siRNA (#s4916, #s4918, Thermofisher), as well as negative control for the experiments. *In vitro* transfections of the oligonucleotides were performed using Lipofectamine 2000 (Invitrogen; Thermo Fisher Scientific, Inc., Waltham, MA, United States) according to the manufacturer’s instructions. After 6 h of transfection, the transfection medium was replaced with a complete medium. All the experiments were carried out at 48 and 72 h post-transfection.

### RNA Extraction and Quantitative Real-Time PCR

To detect the expression of miRNA and mRNA total RNA was extracted using TRIZOL reagent (Invitrogen, Carlsbad, CA, United States) according to the manufacturer’s instructions. cDNA was synthesized from 1 μg of total RNA using a High-Capacity cDNA Reverse Transcription kit (Applied Biosystems) and a TaqMan^®^ MiRNA Reverse Transcription kit (Applied Biosystems, United States). Real-time-qPCR was performed with a TaqMan^®^ Universal Master Mix (Applied Biosystems) and TaqMan^®^ 20× assay (Applied Biosystems) by following the manufacturer’s protocol on a quant-studio 3 and 5 real-time PCR system (Applied Biosystem, United States). The expression data were uniformly normalized to the internal control. For the miRNA expression, the endogenous control was RNU43, and for the gene expression, the endogenous control was GAPDH, and relative gene and mi-RNA expression was quantified using the 2-ΔΔCt method.

### Cell Invasion and Migration Assays

For the migration assay, 5 × 10^4^ cells (72 h post-transfection) were seeded in 200 μl of serum-free medium into the upper chamber of each insert (353097, Corning^®^), and 700 μl of medium supplemented with 10% FBS was added into the lower chamber. For the cell invasion assay, the polyester membranes of the upper surface of the insert (353097, Sigma) were pre-coated with a matrix gel (Corning^®^ Matrigel^®^ Basement Membrane Matrix, Ref: 356234). Following equal amount of above mentioned transfected cells were seeded in 200 μL of serum-free medium on the pre coated insert. The lower chamber was supplemented with 700 μL of complete medium and incubated at 37°C. After 24 h, the cells that invaded and migrated through the membrane were fixed and permeabilized with 70% chilled ethanol for 2 min and with 100% methanol for 15 min, respectively, at room temperature. The invaded and migrated cells were further stained with 0.4% crystal violet for 10 min at room temperature. The cells were then imaged and counted from photographs of five randomly selected fields of the fixed cells.

### Wound Healing Assay

To check the motility capacities of the cells after miR-33b transfection, wound healing assay was performed. At 72 h post-transfection, the cells were seeded in six-well plates to obtain 100% confluence in 24 h. After 24 h, the wound was induced by scratching the monolayer with a micropipette tip, and the dish was placed at 37°C in a 5%-CO_2_ incubator chamber. Pictures were acquired at 0 h and after 24 h using a microscope.

### WST-1 Cell Proliferation

After transfection, cell proliferation was assessed using the WST assay. A total of 3 × 10^3^ transfected cells and negative control cells were seeded in 96-well plates from 1 to 7 days. On each of the mentioned days, cell proliferation was measured using WST-reagent (ab155902, Abcam). Seven percent of the WST reagent was added to each well with phenol red-free media. The plate was incubated for 4 h at 37°C. Then, absorbance was measured at 450 nm in a microplate reader with background correction at 650 nm. The significance of any differences were assessed using *t*-test.

### Cell Cycle Analysis

To analyze cell cycle, 5 × 10^4^ cells were seeded in six-well plates for each condition in triplicates. After 48 h of transfection, the cells were harvested by trypsin and washed with 1× phosphate-buffered saline (PBS) twice. Then, the harvested cells were fixed with chilled 70% ethanol and incubated at −20°C for 6–7 h. The cells were then centrifuged soon after washing with 1× PBS twice, and the pellets were resuspended with propidium iodide (PI) staining buffer (PI/RNase, IMMUNOSTEP) and stored at 4°C overnight. Stained cells were acquired for cell cycle analysis by flow cytometry using a FACSVerse^TM^ flow cytometer (BD Bioscience, United States), and raw data were analyzed by FlowJo software.

### Apoptosis Analysis

Apoptotic cells were determined by double staining using FITC Annexin V Apoptosis Detection Kit with PI (ANXVKF-100T, IMMUNOSTEP) according to the manufacturer’s recommendation. Briefly, 1 × 10^5^ cells were seeded in a six-well plate. After 72 h post-transfection, the supernatant medium was taken in one tube. The attached cells were harvested by trypsinizing and were collected into the same tube. The cells were washed with 1× PBS twice, and the pellet was resuspended with 1× annexin binding buffer. Five microliters of annexin V-FITC and 5 μl of PI were added to the resuspended cells and incubated for 15 min at room temperature in the dark. Furthermore, 400 μl of 1× binding buffer was added with DAPI (0.1 mg/ml, 1–2 μl). The stained cells were acquired for cell cycle analysis by flow cytometry using a FACSVerse^TM^ flow cytometer (BD Bioscience, United States), and raw data were analyzed by FlowJo software.

### Western Blot Analysis

At the indicated time (72 h), the whole lysate of transfected cells was extracted using Thermo Scientific^TM^ RIPA lysis buffer (Ref: 89900). The lysates were transferred to a clean microfuge tube, placed on ice for 30 min, and centrifuged for 30 min at 13,000 rpm. The supernatant was transferred to a fresh microfuge tube, and the protein concentration was determined using a BCA protein assay kit (PierceTM BCA Protein Assay Kit, Ref: 23227). The protein lysates were separated on 10% SDS PAGE and transferred to nitrocellulose membranes (Ref: 1620115, Bio-Rad). The membranes were blocked in 5% BSA for 1 h and then incubated with antibodies of E-cadherin (BD Biosciences, #610181), ß-catenin (BD Biosciences, #610153), vimentin (BD Biosciences, #550513), EZH2 (Cell Signaling, #1674905S), and GAPDH (Thermo Scientific^TM^, #MA5-15738) overnight at 4°C. On the following day, the membranes were washed and subsequently incubated with the appropriate HRP-conjugated secondary antibodies for 1 h at room temperature. Following this incubation, the membranes were washed and briefly incubated with a Pierce^TM^ ECL Western Blotting Substrate western blotting detection reagent (Thermo Fisher Scientific^TM^, Ref: 32106).

### Clinical Samples and RNA Isolations

Formalin-fixed and paraffin-embedded samples of human BC tissues from different subtypes of BC patients and breast samples from healthy donors were selected to analyze the expression of miR-33b and EZH2 gene. The total RNA was isolated from tissue blocks using the RecoverAll Total Nucleic Acid Kit (Ambion) for standard mRNA/miRNA analysis. One microgram of total RNA was retro-transcribed with random primers (for gene expression) and specific primers (for miRNA expression) using Reverse Transcription Kit (Applied Biosystems), and 5 ng of cDNA was used for quantitative PCR for both gene and miRNA expression analysis. The quantitative PCR analysis was performed as mentioned above.

### TCGA Data Analysis

The expression data for miRNA-33b were obtained from Xena browser database^1^ for The Cancer Genome Atlas (TCGA) BC, which contained 1,285 cases of different BC subtypes solid tumors and normal. From there, we were able to obtain only 211 specimens with clinical details, including luminal B (*n* = 49), basal-like (*n* = 26), luminal A (*n* = 92), HER2+ (*n* = 18), and normal solid tissue (*n* = 26). For EZH2 expression, we used the same data base, which contained 1,248 cases of different BC subtypes solid tumors and normal, wherefrom we only obtained 522 specimens with clinical details, including luminal B (*n* = 127), basal-like (*n* = 98), luminal A (*n* = 231), HER2 + (*n* = 58), and normal solid tissue (*n* = 8). The statistical analysis was done using Shapiro–Wilk normality test, and based on normality test results, parametric and non-parametric tests were applied to obtain the *p* value of the analysis.

### *In silico* Survival Analysis

Overall survival associated with miRNA and gene expression was analyzed using Kaplan–Meier plotter (KM plotter) tool^2^. This tool works upon a database containing different subtypes of BC Affymetrix microarray samples and associated survival information, with a median follow-up of 120 months. Based on METABRIC dataset, by specifying the miRNA name and the gene name on the search tool and filtering down to “all breast cancer subtypes and HER2+ subtype,” the survival rates according to miRNA or gene expression were obtained. The hazard ratio (HR) with 95% confidence intervals and log-rank *p*-value were calculated and shown. The obtained results were used to identify the prognostic value of miR-33b and EZH2 expressions on HER2+ BC.

### Statistical Analysis

The sample and the control groups were compared using two-tailed Student’s *t*-test. All data presented include median and standard deviation. *P*-values less than 0.05 were considered to be statistically significant.

### Ethical Approval

The study was conducted in accordance with recognized ethical guidelines (Declaration of Helsinki), and it was approved by the INCLIVA institutional review board (protocol number: 2018/077). All the participants in the study signed a written informed consent.

## Results

### Expression of miR-33b and EZH2 in HER2+ Breast Cancer Cell Lines, Patient Samples, and Non-tumorigenic Cells

**FIGURE 1:**
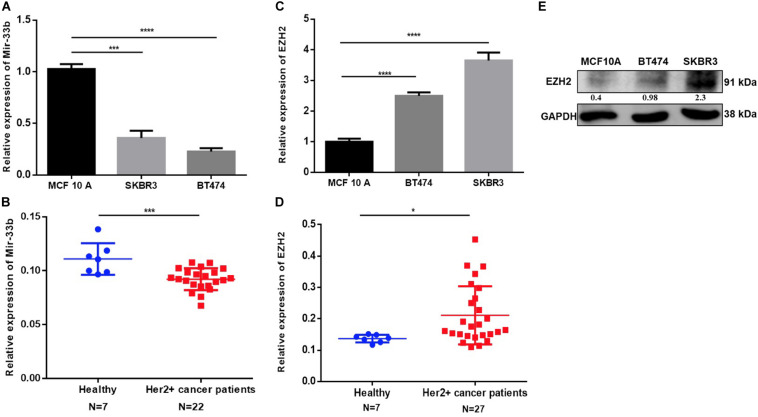
Expression of miR-33b and EZH2 in HER2+ cancer cell lines, cancer patients, and non-tumorigenic cells. The relative expression of miR-33b was determined in HER2+ cancer cell lines and HER2+ tumor tissues, non-tumorigenic epithelial cell line, and healthy controls by Q-PCR and normalized to RNU43 (for cell lines) and miR-16 (for tissue) **(A,B)**. EZH2 expression was determined also in the same specimen by Q-PCR and normalized to GAPDH (for cell lines), PPIA, and MRPL19 (for tissue) **(C,D)**. EZH2 protein expression was determined by western blot in HER2 + and MCF-10A cell line **(E)**. Student’s *t*-test was used to analyze the significant differences. *p < 0.05, ***p < 0.001, ****p < 0.0001.

MiR-33b expression was determined in four human BC cell lines, including MDA-MB-468, MCF-7, BT474, and SKBR3 (HER2+), with the non-tumorigenic epithelial cell lines MCF12A and MCF-10A as controls. The quantitative PCR (Q-PCR) data revealed that miR-33b expression was significantly higher in MCF-10A as compared to that in HER2+ BC cell lines (Figure 1A). An analysis of tissue samples from Department of Oncology, Hospital Clinico de Valencia and the TCGA database for a HER2+ BC retrospective cohort confirmed a significantly lower miR-33b expression level than the breast control samples (Figure 1B and Supplementary Figure 1E). The EZH2 expression was significantly higher in HER2+ BC cell lines than in MCF-10A as determined at the mRNA level (Figure 1C) and at the protein level (Figure 1E). Similarly, a significantly higher EZH2 expression was found in HER2+ BC tissue samples vs. healthy breast tissues (Figure 1D) and also from the TCGA data portal (Supplementary Figure 1F). The analysis of miR-33b and EZH2 in other BC subtypes showed as well a significant higher expression of miR-33b in control cell lines *vs* cancer cell lines and an oppositely significant higher expression of EZH2 on cancer cell lines in comparison with those of the controls (Supplementary Figures 1A,C). MiR-33b and EZH2 expression in the TNBC patients’ samples showed the same tendency than the HER2 + samples compared with those in healthy breast tissues (Supplementary Figures 1B,D). These data altogether suggested a downregulation of miR-33b and a high expression of EZH2 in HER2+ BC subtypes both *in vitro* and in BC tissues, being one of the important reasons for the high aggressiveness of this subtype.

### Overexpression of miR-33b Reduces Proliferation and Induces Apoptosis in HER2+ BC Cell Lines

**FIGURE 2:**
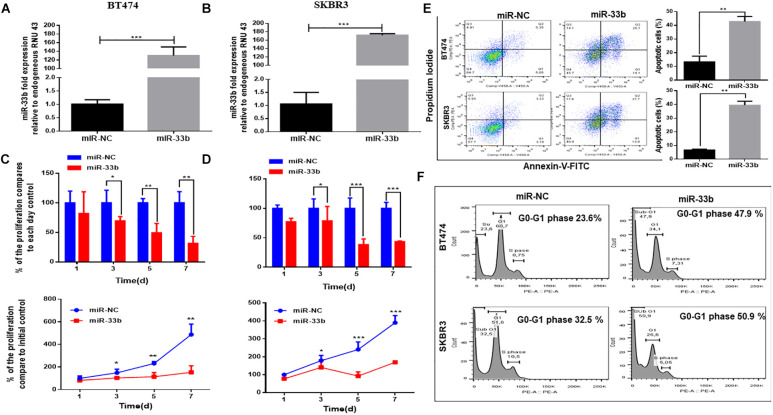
MiR-33b reduces cell proliferation and induces early and late apoptosis. Satisfactory transfection efficiency of miR-33b mimic in both HER2 + cell lines: BT474 **(A)** and SKBR3 **(B)**. Cell proliferation was carried out through WST assay in both cell lines after transfection with miR-33b mimic until 7 days **(C,D)**. The apoptotic cell population was determined at 72 h in both cell lines by flow cytometry through annexin-V/PI staining **(E)**. To confirm the apoptotic population and the percentage of sub-G0/G1 population, cell cycle was analyzed through PI-RNAse assay at 48 h **(F)**. Student’s *t*-test was used to analyze the significant differences. *p < 0.05, **p < 0.01, ***p < 0.001.

To determine the potential effect of miR-33b on cell proliferation and apoptosis in HER2 + BC, cells were transfected with miR-33b mimic or scramble miR (miR-NC). Its expression was confirmed by Q-PCR in both cell lines (Figures 2A,B). The WST cell proliferation assay was carried out to observe the proliferation effect, which showed that the overexpression of miR-33b significantly decreased cell proliferation as compared to scramble in BT474 and SKBR3, and the inhibitory effects showed a statistical significance after 7 days (Figures 2C,D). A recent study showed that miR-33b regulates cell cycle and apoptosis (23). To confirm this effect in our model, we evaluated apoptosis by annexin-V. As shown in Figure 2E, the ectopic expression of miR-33b induced early and late apoptosis in both HER2+ cell lines. To verify these results, we further investigated the cell cycle by PI/RNAse with miR-33b transfected cell lines, which showed a considerable increase of cells in the sub-G0/G1 phase compared to the control and a reduction almost by half in the number of cells in G1 and S phases (Figure 2F). Collectively, it showed that miR-33b has an anti-proliferative effect on HER2+ BC cell lines and induced apoptosis with arrest of the cells at sub-G0/G1 phase.

### Overexpression of miR-33b Suppresses Invasion, Migration, EMT Process, and Expression of EZH2 in HER2+ BC Cell Lines

**FIGURE 3:**
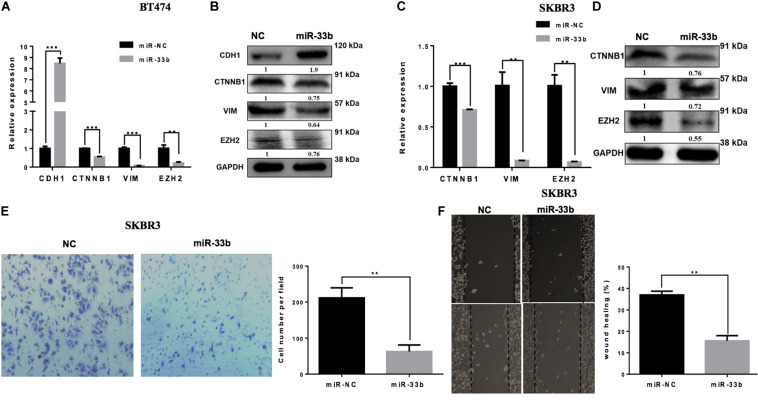
miR-33b inhibits cell epithelial–mesenchymal transition, migration, and invasion in HER2+ cell lines. The relative expression of the EMT genes and protein expression were checked after transfection with miR-NC and miR-33b in HER2+ cell lines: BT474 **(A,B)** and SKBR3 **(C,D)**. SKBR3 cells were transfected with miR-33b mimic, and cells penetrating the membrane were fixed with 0.4% crystal violet stain after 24 h to evaluate the invasion capacity **(E)**. A wound healing assay was performed on SKBR3 transfected with miR-33b to explore the migration properties of the cells. Black arrows indicate the wound edge. The relative scratch gap was calculated as the percentage (%) of the remaining scratch gap at the given time point and the original gap at 0 h **(F)**. Student’s *t*-test was used to analyze the significant differences. **p < 0.01, ***p < 0.001.

Tumor cell invasion and metastasis are tightly correlated with various processes, including EMT. During EMT, epithelial cells acquire mesenchymal characteristics with a high expression of vimentin and ß-catenin, whereas the epithelial protein marker CDH1 is downregulated. It has also been described that miR-33b is a key regulator of MYC pathway, and one of the downstream targets of this pathway is EZH2, which is a potential regulator of cell proliferation, EMT, invasion, migration, and drug resistance (24). The overexpression of miR-33b in BT474 induced a statistically significant increase of the expression of CDH1 and significant decreases of ß-catenin, vimentin, and EZH2 (Figure 3A). Consistent results were obtained with SKRB3 (Figure 3C). To confirm these data at the protein level, western blot was performed (Figures 3B,D). However, CDH1 was unable to detect SKBR3 because of its homozygous deletion of a large portion of the gene in this cell line (25). Additionally, migration and invasion assays were planned to explore the anti-metastatic effect of miR-33b. The SKBR3 cells were transfected with miR-33b mimic for 72 h and seeded on matrigel-based transwells to check the invasion capacity within 24 h. The expression of the mature miR-33b was confirmed by Q-PCR in that cell line (data not shown). The results showed that the overexpression of miR-33b induced a decrease in SKBR3 invasion capability compared to the controls (Figure 3E). The migration process was carried out by the wound healing assay. The results showed that miR-33b overexpression significantly reduced the migration properties of HER2+ cells compared to the negative control (Figure 3F). Taken together, these results suggested that miR-33b inhibits cell invasion and migration and acts as a possible crucial regulator of the EMT process in HER2+ BC. Probably it can be an indirect tumour aggressiveness inhibitory effects through targeting EZH2 in the specific BC subtype.

### Inhibiting miR-33b Expression Induces Cell Proliferation, Invasion, and Migration in Non-tumorigenic Cells

**FIGURE 4:**
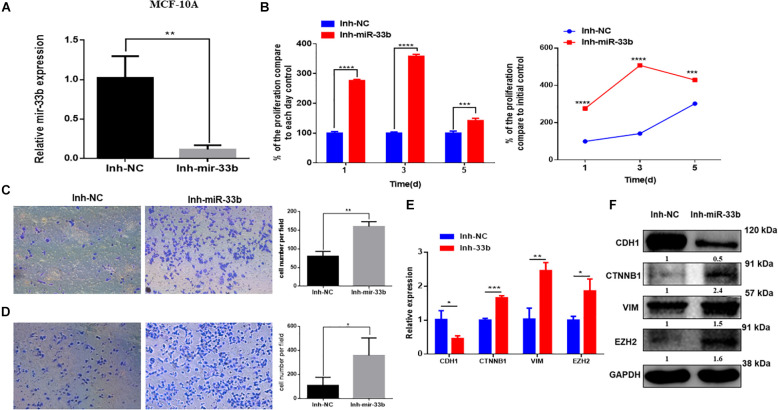
Inhibiting miR-33b induces cell proliferation, migration, invasion, and epithelial–mesenchymal transition (EMT) markers in non-tumorigenic breast cell lines. Satisfactory transfection efficiency of miR-33b inhibitor in MCF-10A **(A)**. Cell proliferation was carried out through WST assay in MCF-10A after transfection with miR-33b inhibitor until 5 days **(B)**. Reducing the expression of miR-33b increased the invasive **(C)** and the migration **(D)** abilities of MCF-10A. mRNA **(E)** and protein **(F)** expression of the EMT markers using Q-PCR and western blot, respectively. Student’s *t*-test was used to analyze the significant differences. *p < 0.05, **p < 0.01, ***p < 0.001, ****p < 0.0001.

Because the overexpression of miR-33b reduced cell proliferation, invasion, migration, and EMT in HER2 + BC cell, we wondered if inhibition of this miRNA in control cells would have the opposite effect. To further investigate it, the miR-33b inhibitor was transfected in control cell lines. The efficiency of transfection was confirmed at 72 h post-transfection in both non-tumorigenic cell lines (Figure 4A and Supplementary Figure 2A). WST assay was carried out to evaluate the effect in proliferation, which showed that inhibition of miR-33b increases the proliferation at 3 days as well as at 5 days in both control cell lines (Figure 4B and Supplementary Figure 2B). After 72 h of transfection with the miR-33b inhibitor, the cells were seeded on transwells to evaluate the invasion (Figure 4C and Supplementary Figure 2C) and the migration (Figure 4D and Supplementary Figure 2D) properties of the cells. The results showed that inhibition of miR-33b significantly promoted cell migration and invasion in both non-tumorigenic cell lines. These data suggested that miR-33b is required to control cell migration, invasion, and proliferation.

### Downregulation of miR-33b Induces EMT and EZH2 Expression

To better understand the molecular mechanism of action of miR-33b on cell migration and invasion, we opted to explore the regulation of EMT signaling and the regulation of EZH2. Control cell lines were transfected with an inhibitor of miR-33b, and at 72 h after transfection, EMT signaling pathway factors were checked on the level of mRNA and protein expression. The results showed that, with the inhibition of miR-33b, the expression of CDH1 was significantly diminished, and there was an increase of ß-catenin, vimentin, and EZH2 in both mRNA and protein level (Figures 4E,F and Supplementary Figures 2E,F). Thus, it supports that miR-33b can regulate EMT signaling in both control and cancer cell lines.

### Downregulation of EZH2 Inhibits Proliferation in HER2+ BC Cell Lines

**FIGURE 5:**
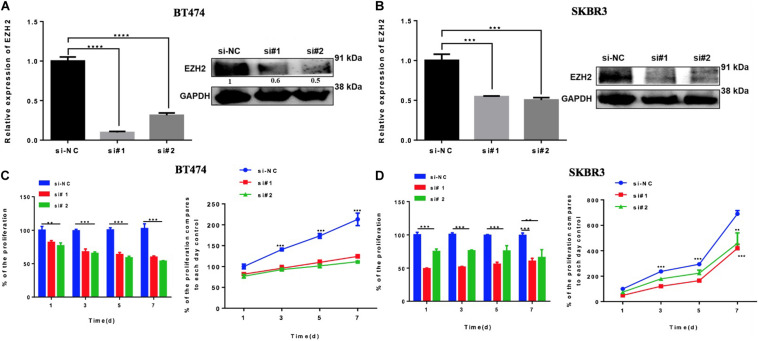
Reducing EZH2 expression induces proliferation in HER2 + cell lines. Q-PCR and Western blot results showing the gene silencing efficiency of siRNA sequences targeting EZH2 using two silencers in BT474 **(A)** and SKBR3 **(B)** cell lines. Cell proliferation was carried out through WST assay in both cell lines, BT474 **(C)** and SKBR3 **(D)**, after transfection with two different silencers until 7 days. Student’s *t*-test was used to analyze the significant differences. **p < 0.01, ***p < 0.001, ****p < 0.0001.

The previous results suggest that miR-33b is regulating EZH2. In order to evaluate the role of EZH2 in HER2+ BC cell lines, BT474 and SKBR3 cells were transfected with two different si-EZH2. Confirmation of gene and protein silencing was performed by Q-PCR and by western blot, respectively. Both siRNAs significantly inhibited EZH2 expression in BT474 and SKBR3 cells compared to controls (Figures 5A,B). To explore the effect of silencing of EZH2 on cancer cell proliferation, the WST assay showed that a lower expression of EZH2 significantly decreased the cell proliferation in BT474 and SKBR3, and this inhibitory effect showed statistical significance until 7 days (Figures 5C,D). These results indicated that EZH2 may act as a crucial gene for tumor aggressiveness in HER2+ BC through modulating cell proliferation.

### Downregulation of EZH2 Inhibits EMT, Invasion, and Migration in HER2+ BC Cell Lines

**FIGURE 6:**
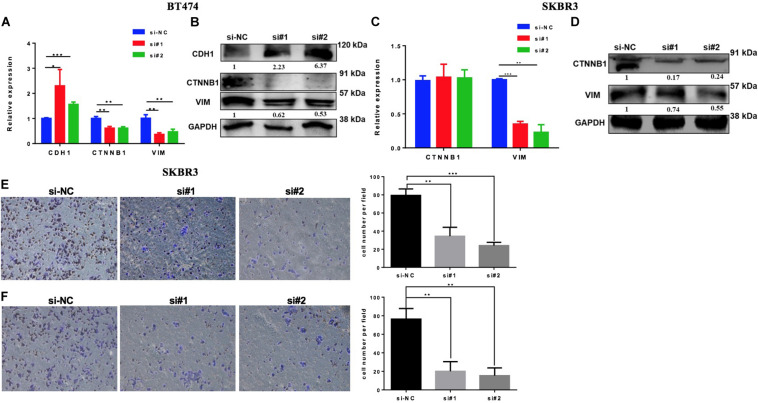
Silencing EZH2 inhibits epithelial–mesenchymal transition (EMT) markers, invasion, and migration in HER2+ breast cancer cells. Q-PCR and western blot results showing the EMT marker levels after silencing EZH2 in BT474 **(A,B)** and SKBR3 **(C,D)**. Reducing the expression of EZH2 inhibits the invasive **(E)** and the migration **(F)** abilities of SKBR3 cell. Student’s *t*-test was used to analyze the significant differences. *p < 0.05, ** *p* < 0.01, ***p < 0.001.

High levels of EZH2 have been involved in BC progression by the regulation of the EMT process. To evaluate if the downregulation of EZH2 mediates the inhibition of the EMT pathway in our model, we silenced the EZH2 in BT474 and SKBR3 by two different siRNAs to analyze the gene set enrichment of EMT. Both cell lines were transfected with two specifically different siRNAs of EZH2. The downregulation of the EMT genes was confirmed by Q-PCR and the protein expression was evaluated by western blot in both cell lines compared to the control (Figures 6A–D). The results showed that the downregulation of EZH2 expression induced a statistically significant increase of CDH1 and a decrease of ß-catenin and vimentin in BT474 at both the mRNA and the protein levels. There were no changes in ß-catenin (CTNNB1) at the mRNA level in SKBR3 with the silencing of EZH2. However, at the protein level, there was a reduction in the expression of ß-catenin and vimentin at both the gene and the protein levels. In addition, the silencing of EZH2 affects invasion and migration, resulting in a decrease in the SKBR3 cell line (Figures 6E,F). Altogether these results showed that EZH2 induces EMT to promote invasion and migration in HER2 + BC cells.

### High miR-33b Expression Levels Were Correlated With Favorable Overall Survival Outcome in HER2+ BC Patients

**FIGURE 7:**
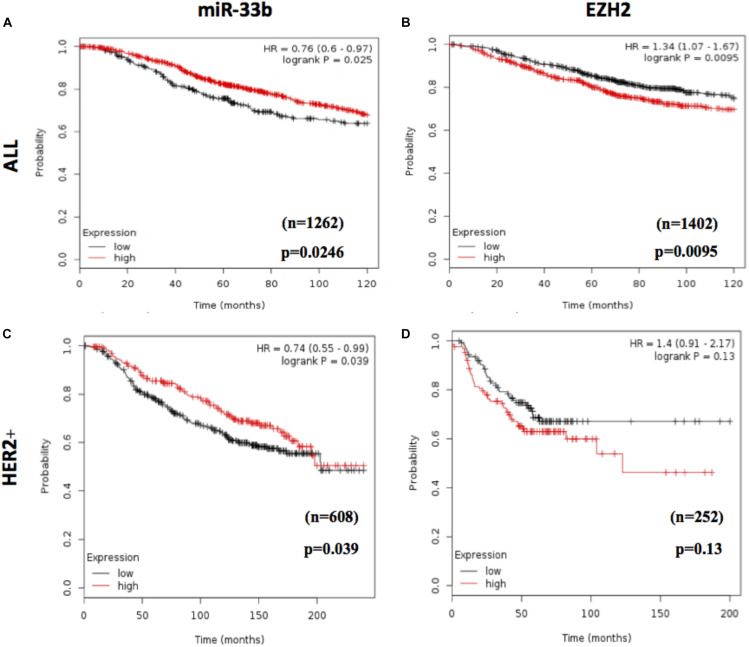
Prognostic value of miR-33b and EZH2 expression in breast cancer patients. *In silico* overall survival analysis of miR-33b **(A)** or EZH2 **(B)** in breast cancer (BC) patients with Kaplan–Meier plotter. Survival analysis in a cohort of HER2+ BC patients: miR-33b **(C)** or EZH2 **(D)**.

To assess the prognostic value of miR-33b and EZH2, we used an *in silico* survival analysis of BC patients with the Kaplan–Meier plotter. As a result, the BC patients with high miR-33b expression showed a statistically significant improvement in overall survival (OS) (*p* = 0.0246, HR = 0.76, 95% CI 0.60–0.97) (Figure 7A), suggesting a good prognostic role of this miRNA. The, opposite results were found with EZH2 when it was evaluated among the same set of patients; it was observed that a high expression was associated with a worse OS (*p* = 0.0095, HR = 1.34, 95% CI 1.07–1.67) (Figure 7B). Similar results were obtained in a cohort of HER2 + BC patients. We found that a high miR-33b expression maintained a significant–good prognosis in terms of OS (*p* = 0.039, HR = 0.74, 95% CI 0.55–0.99) (Figure 7C). At the same time, high EZH2 expression showed a poor prognosis (HR = 1.4, *p* = 0.13). Nevertheless, it was not statistically significant, probably due to the low number of subjects (Figure 7D). These results corroborated the importance of this axis as a prognostic factor in HER2+ BC.

## Discussion

A lot of accumulated data have pointed out that several miRNAs drive tumorigenesis and drug resistance and suppress cancer progression by targeting different oncogenes (26). Although multiple studies have been carried to study the roles of miRNAs in BC, most of them have focused on BC in general and not on the specific subtypes. HER2+ BC subtype is one of the cancers with a worse prognosis and is associated with inferior outcomes in survival (27), being an entity with a large heterogeneity at multiple levels (28). In recent studies, miRNAs are being identified as one of the key regulators to uncover the molecular mechanisms of the heterogeneity in HER2 + BC.

The miR-33 family is one of the highly conserved miRNA families that consists of two members: miR-33a and miR-33b (29). They both act as a tumor suppressor in different cancers such as non-small cell lung cancer (30), TNBC (31), esophageal squamous cell carcinoma (32), and colorectal cancer (33) *via* targeting EMT and proliferation. For the first time in this study, we reported that miR-33b was downregulated in breast HER2+ tumor samples when compared to normal breast tissues and that the under-expression of miR-33b is related to a poor prognosis in HER2+ patients. We also found that miR-33b expression was higher in normal breast epithelial cell lines than in HER2+ BC cell lines. It has been described as well that, in TNBC, miR-33b represses cancer progression and metastasis by targeting oncogenes like *SALL4*, *TWIST1*, and *HMGA2* (13). These data indicate that miR-33b acts as an onco-suppressive miRNA in BC progression. To investigate the specific mechanism of miR-33b in HER2+ BC progression, miR-33b was ectopically overexpressed in different HER2+ cell lines, where it was shown that the upregulation of this miRNA inhibits cancer cell invasion and migration. As it has been already reported that this miRNA regulates EMT (34), we here demonstrated that the overexpression of miR-33b inhibits the EMT process in HER2+ subtype of BC by regulating EZH2 expression. Furthermore, we reported that the overexpression of miR-33b has an impact on cell proliferation and induces apoptosis in this BC subtype. Besides that, this miRNA also arrests the cell cycle in the sub-G0/G1 phase as compared with the other phases, which is in concordance with previous results in lung cancer (8). Recently, some authors suggested that miR-33a can regulate EZH2 by their direct interaction (21). We checked *in silico* the physical interaction between miR-33b and EZH2 in Targetscan, miRDB-MicroRNA Target Prediction Database, miRNet, miRTarBase, miRanda databases, and the Freiburg RNA tools. In the latter case, we found an interaction with a yield of very low energy. Based on this information, we performed the luciferase assay and we found that there is no such direct interaction between miR-33b and EZH2 (data not shown), which clarified that although miR-33a and miR-33b belong to the same family, they regulate the same gene in a different way. It has been previously demonstrated that MYC binds to the EZH2 promoter and directly activates its transcription (20). Besides that, EZH2 expression is positively correlated with MYC expression in prostate cancer (35). Moreover, it has been already described that MYC is a direct target of miR-33b (10). Thus, in our present study, we showed that the ectopic overexpression of miR-33b regulates MYC in our models (Supplementary Figures 3A–D) and the sequences of miR-33b have binding sites within the human MYC 3′UTRs (TargetScan can, Supplementary Figure 3E). Considering all these, we suggest EZH2 as a target of miR-33b *via* regulating the MYC (Supplementary Figure 3F).

Epithelial–mesenchymal transition is a crucial process during the development of tumorigenesis and metastasis. Enormous evidences indicate that EMT is responsible for cancer cell invasion and migration and an initial step of metastasis. *EZH2* is reported to be upregulated in aggressive BC (36) and involved in epigenetic, post-translational modifications and EMT program by suppressing CDH1 expression (37). In nasopharyngeal carcinoma, miR-142-3p was downregulated by DNA methylation due to EZH2’s recruitment of DNMT1 which occupied the upstream region of the miR-142 and determined ZEB2 activation, leading to EMT and metastasis (38). Furthermore, EZH2 is a direct target of miR-26a in docetaxel resistance cells, which could significantly suppress proliferation, facilitate apoptosis, inhibit the metastasis ability, and reverse EMT to mesenchymal–epithelial transition in lung adenocarcinoma cells (39). In oral tongue squamous cell carcinoma, miR-101 inhibits the expression of EZH2 *via* two transcription factors, Snail and Slug (40). In BC, miR-92b may negatively regulate the expression of EZH2, promoting autophagy and decreasing tumor cell viability, migration, and invasion (41). Additionally, miR-139-5p transcription is inhibited by EZH2 through upregulating H3K27me3; thereby, the downregulation of EZH2 and the upregulation of miR-139-5p impede EMT in lymph node metastasis pancreatic cancer (42). Accumulating all these summarized results, the expression of EZH2 is upregulated in different types of cancer, and its inhibition is required by different miRNAs and drugs to reduce cancer progression. Given that the behavior of EZH2 is context dependent, in this study we investigated the role of EZH2 specifically in HER2+ BC. In our study, we determined that EZH2 is highly expressed in HER2+ BC cell lines as well as in solid tumors in comparison with normal epithelial cell line and normal breast tissue which show an inverse correlation. To dig more on the molecular mechanisms of EZH2, it has been silenced through two different silencers in BC cell lines, which resulted in the inhibition of cell proliferation, migration, invasion, and EMT in HER2+ BC cells, confirming that EZH2 expression has a crucial role in HER2+ BC progression (Supplementary Figure 3F). Future *in vivo* experiments to evaluate the role of miR-33b in HER2+ BC metastasis are needed.

In summary, EZH2 might be an important factor of HER2+ BC progression and associated with a decrease in the overall survival of patients since EMT has been critically discussed as the key process in tumor aggressiveness and metastasis (43). Our findings in the present study demonstrate for the first time that miR-33b acts as a suppressive miRNA in HER2+ BC, which could inhibit tumor migration and invasion partly by impeding EMT through the repression of the MYC–EZH2 loop. This study suggests a novel miR-33b/MYC/EZH2 axis that modulates the growth and the progression of breast cells and could be clinically useful to design new drugs against HER2+ subtype cancer.

## Data Availability Statement

All datasets generated for this study are included in the article/Supplementary Material.

## Ethics Statement

The studies involving human participants were reviewed and approved by INCLIVA institutional review board (protocol number: 2018/077). The patients/participants provided their written informed consent to participate in this study.

## Author Contributions

AL and PE contributed to the conceptualization and design of the study. BPa, IG-C, AA-A, and ET developed the methodology. BPi, PC, AA-A, and EA contributed to the acquisition of data. OB, ET, BB, JC, and PE contributed to the analysis and interpretation of data. PE, BPa, IG-C, JA, FR, AL, AR, and JC contributed to the writing, review, and/or revision of the manuscript. EA, BB, CH, MM, and OB provided administrative, technical, or material support. JC, AL, and PE supervised the study. All authors contributed to the article and approved the submitted version.

## Conflict of Interest

JA and AL report being advisory board members from Roche. JA and AL report receiving other honoraria from Roche as speaker’s bureau or travel grants. The remaining authors declare that the research was conducted in the absence of any commercial or financial relationships that could be construed as a potential conflict of interest.

## Footnotes

1 https://xenabrowser.net/

2 http://kmplot.com/analysis/
