# Supplementary material for: MicroRNA-33b Suppresses Epithelial–Mesenchymal Transition Repressing the MYC–EZH2 Pathway in HER2+ Breast Carcinoma
Source: Front Oncol. 2020 Sep 10;10:1661. doi: 10.3389/fonc.2020.01661 (PMC7511588; doi:10.3389/fonc.2020.01661)
Supplement: Supplementary file 1 [file Data_Sheet_1.docx]

Supplementary Material

**Supplementary Figure 1. Expression of the miR-33b and EZH2.** The relative expression of the miR-33b (A) and EZH2 (C) was determined in different cancer cell lines and non-tumorigenic epithelial cell lines by Q-PCR and normalized to RNU43 and GAPDH respectively. Relative expression of the miR-33b (B) and EZH2 (D) was determined by Q-PCR in TNBC cancer tissue and healthy control. TCGA database analysis confirms the expression of miR-33b and EZH2 in different subtypes of breast cancer tissue compared to normal solid tissue (E,F). Student's t-test was used to analyse the significant differences. *p<0.05, ** p<0.01, ***p<0.001, ****p<0.0001

**Supplementary Figure 2: Inhibiting miR-33b induces proliferation, migration, invasion and EMT markers in the non–tumorigenic MCF-12A cell line.** Satisfactory transfection efficiency of miR-33b inhibitor in MCF-12A (A). Cell proliferation was carried through WST assay after transfection with miR-33b inhibitor until 5 days (B). Reducing expression of miR-33b increased the invasive (C) and migration (D) abilities of MCF-12A. mRNA (E) and protein (F) expression of the EMT markers using Q-PCR and western blot respectively. Student's t-test was used to analyze the significant differences. *p<0.05, **p<0.01, ***p<0.001.

**Supplementary Figure 3**. **MiR-33b inhibiting EZH2 through MYC inhibition.**  Overexpression of miR-33b in BT474 (A) and SKBR3 (B) HER2+ BC cell lines reduces the expression of MYC determined by Q-PCR and Western-blot. Contrary, the inhibition of miR-33b in MCF-10A (C) and MCF-12A (D) control breast cell lines increase the expression of MYC determined by Q-PCR and Western-blot. Binding of the sizes of the sequence of miR-33b within the human MYC 3′UTRs using Targetscan (E). Hypothetic mechanism of action, suggesting EZH2 as a target of miR-33b via regulating MYC (F). Student's t-test was used to analyze the significant differences. *p<0.05, **p<0.01, ***p<0.001.
